# EphB4 as a Novel Target for the EGFR-Independent Suppressive Effects of Osimertinib on Cell Cycle Progression in Non-Small Cell Lung Cancer

**DOI:** 10.3390/ijms22168522

**Published:** 2021-08-07

**Authors:** Ren Nanamiya, Ryoko Saito-Koyama, Yasuhiro Miki, Chihiro Inoue, Teeranut Asavasupreechar, Jiro Abe, Ikuro Sato, Hironobu Sasano

**Affiliations:** 1 Department of Pathology, Tohoku University Graduate School of Medicin, Sendai 980-8575, Japan; ren.nanamiya.p5@dc.tohoku.ac.jp (R.N.); miki@patholo2.med.tohoku.ac.jp (Y.M.); chihiro_inoue@med.tohoku.ac.jp (C.I.); teeranut_NAN1209@hotmail.com (T.A.); hsasano@patholo2.med.tohoku.ac.jp (H.S.); 2 Department of Thoracic Surgery, Miyagi Cancer Center, Natori 981-1293, Japan; abe-ji390@miyagi-pho.jp; 3 Department of Pathology, Miyagi Cancer Center, Natori 981-1293, Japan; sato-ik510@miyagi-pho.jp

**Keywords:** osimertinib, EphB4, cell cycle, prognostic factor, predictor of therapeutic effect

## Abstract

Osimertinib is the latest generation epidermal growth factor receptor (EGFR)-tyrosine kinase inhibitor used for patients with EGFR-mutated non-small cell lung cancer (NSCLC). We aimed to explore the novel mechanisms of osimertinib by particularly focusing on EGFR-independent effects, which have not been well characterized. We explored the EGFR-independent effects of osimertinib on cell proliferation using NSCLC cell lines, an antibody array analysis, and the association between the action of osimertinib and the ephrin receptor B4 (EphB4). We also studied the clinicopathological significance of EphB4 in 84 lung adenocarcinoma patients. Osimertinib exerted significant inhibitory effects on cell growth and cell cycle progression by promoting the phosphorylation of p53 and p21 and decreasing cyclin D1 expression independently of EGFR. EphB4 was significantly suppressed by osimertinib and promoted cell growth and sensitivity to osimertinib. The EphB4 status in carcinoma cells was positively correlated with tumor size, T factor, and Ki-67 labeling index in all patients and was associated with poor relapse-free survival in EGFR mutation-positive patients. EphB4 is associated with the EGFR-independent suppressive effects of osimertinib on cell cycle and with a poor clinical outcome. Osimertinib can exert significant growth inhibitory effects in EGFR-mutated NSCLC patients with a high EphB4 status.

## 1. Introduction

Lung cancer is one of the most common causes of cancer-related deaths worldwide [1]. In lung adenocarcinoma, the most frequent histological subtype among non-small cell lung cancer (NSCLC), molecular-targeted therapy for various driver mutations such as the epidermal growth factor receptor (EGFR) and anaplastic lymphoma kinase (ALK) have been recently used in clinical settings to provide better clinical outcomes. Among the therapeutic relevant mutations, EGFR mutations account for approximately 50% of lung adenocarcinoma cases in East Asia [2], and EGFR-tyrosine kinase inhibitors (TKIs) such as gefitinib, erlotinib, and afatinib have been used as molecular-targeted therapeutic agents for patients with EGFR mutation-positive lung adenocarcinoma. These agents have contributed to the improvement of clinical outcomes [3,4,5,6,7,8], but therapeutic resistance is inevitable within several months of their administration [9]. The most common cause of resistance is the T790M mutation, in which threonine, the 790th amino acid of exon 20 of the EGFR gene, replaces methionine. The T790M mutation accounts for more than half of the causes of acquired resistance [9,10]. Therefore, to overcome this resistance, novel EGFR-TKIs such as osimertinib have been developed [11,12,13]. Osimertinib can exert antitumor effects on lung adenocarcinoma harboring a T790M mutation by binding to a completely different site of EGFR compared to other EGFR-TKIs. In addition, the results of the FLAURA study have also demonstrated that osimertinib provides a better prognosis than gefitinib and erlotinib as a first-line treatment for patients with EGFR mutation-positive lung adenocarcinoma [14]. Therefore, osimertinib is becoming the gold standard for the treatment of patients with EGFR mutation-positive lung adenocarcinoma, and its use is expected to expand in near future.

EGFR-dependent pathways of EGFR-TKIs, including gefitinib, erlotinib, and osimertinib, have been well studied, and common mechanisms between them that exert antitumor effects in EGFR mutation-positive lung adenocarcinoma have been demonstrated [15,16,17]. Although the EGFR-independent antitumor effects of these agents have been reported in leukemia and squamous lung cancer patients [18,19], only a few studies have evaluated their effects on lung cancer patients [20]. In the aforementioned studies, the EGFR-independent effects of osimertinib were attributed to the inhibition of human EGFR-related 2 (HER2) phosphorylation and lysine specific demethylase 1 and to the induction of autophagy [21,22,23]. Osimertinib has also been reported to inhibit not only EGFR and HER2 but also various kinases such as ALK and fibroblast growth factor receptors [11]. However, the details of the EGFR-independent mechanisms of osimertinib are unknown.

In this study, we focused on the Ephrin receptor (Eph) B4 as one of the key molecules forthese mechanisms based on our results, as described below. EphB4 is a member of the Eph family and consists of 14 types of receptors in humans [24]. Ephs are activated by binding to the ephrin ligand expressed on the cell membrane and to other Ephs located on the cell membrane [25]. Ephs regulate various intracellular signaling systems, particularly signals mediated by low molecular weight G proteins of the Ras/Rho family, which regulate the construction of actin filaments and result in changes in cell morphology and decreased adhesion [25,26]. Various pathways, such as phosphoinositide 3-kinase, Ras, Rho, Abl pathways, and downstream Ephs have been reported to play an important role in cell proliferation, infiltration, metastasis, and angiogenesis [27,28,29]. In addition, EphB4 was also reported to be a poor prognostic factor in tumors such as glioblastoma and breast, ovarian, and bladder cancer [30,31,32,33]. According to these reports, EphB4 was reported to increase cell viability via the phosphatidylinositol 3-kinase/Akt signaling pathway [31,34].

Therefore, in this study, we attempted to explore novel factors possibly associated with the EGFR-independent mechanisms of osimertinib in lung cancer. In particular, we studied the correlation between osimertinib therapy and EphB4, which was identified by in vitro analysis in NSCLC to establish new therapeutic effect predictors of osimertinib.

## 2. Results

### 2.1. Osimertinib Significantly Inhibited Cell Proliferation of NSCLC Cells

**Figure 1:**
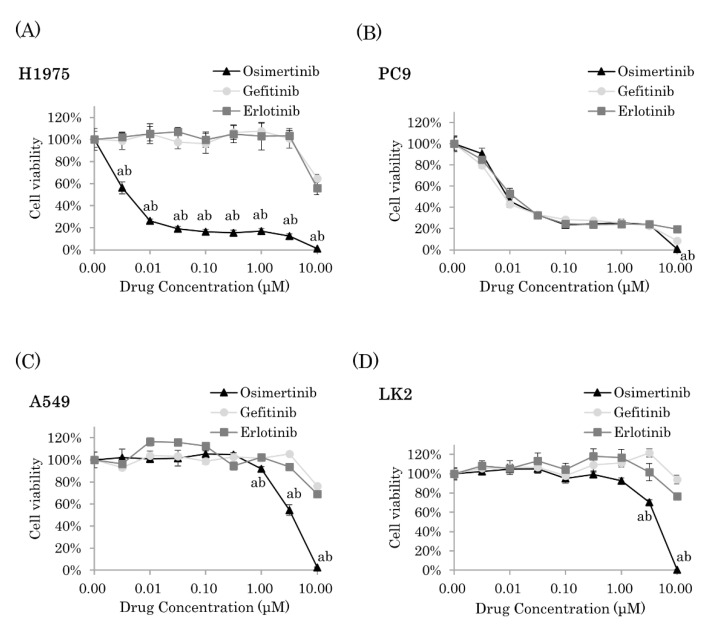
The inhibitory effects of EGFR-TKIs on NSCLC cell proliferation. NSCLC cell lines ((**A**), H1975; (**B**), PC9; (**C**), A549; and (**D**), LK2) were treated with erlotinib, gefitinib, and osimertinib for 72 h at various concentrations (*n* = 6). The results were expressed as mean ± standard deviation. a: Osimertinib vs. Gefitinib, *p* < 0.05. b: Osimertinib vs. Erlotinib, *p* < 0.05.

We examined the cell viability of four cell lines (H1975, PC9, A549, and LK2) after treatment with EGFR-TKIs (osimertinib, gefitinib, and erlotinib) for 72 h. H1975 cells demonstrated a resistance to gefitinib and erlotinib but showed a sensitivity to osimertinib (Figure 1A). In PC9 cells, all three EGFR-TKIs significantly suppressed cell proliferation; however, at high dose concentrations (10 µM), osimertinib exhibited significantly stronger inhibitory effects on cell proliferation than the other two EGFR-TKIs (gefitinib and erlotinib, *p* < 0.0001) (Figure 1B). In A549 and LK2 cells, osimertinib significantly inhibited cell proliferation at 3 µM (*p* < 0.0001 and *p* = 0.0002, respectively) and at 10 µM (*p* < 0.0001) (Figure 1C,D). In addition, osimertinib exhibited significantly stronger inhibitory effects on cell proliferation than both gefitinib and erlotinib (A549 cells at 1 µM, *p* = 0.0210 and *p* = 0.0016, respectively; at 3 µM, *p* < 0.0001; at 10 µM, *p* < 0.0001 and LK2 cells at 3 µM, *p* < 0.0001 and *p* = 0.0021, respectively; at 10 µM, *p* < 0.0001) (Figure 1C,D). Among these concentrations, the nearest concentration to calculated 50% inhibitory concentration (IC50) was applied as IC50 in this study: for A549, osimertinib at 3µM, gefitinib at 10 µM, and erlotinib at 10 µM; for LK2, osimertinib at 3µM, gefitinib at 10 µM, and erlotinib at 10 µM; for PC9, osimertinib at 10 nM, gefitinib at 10 nM, and erlotinib at 10 nM; for H1975, gefitinib at 10 µM and erlotinib at 10 µM.

### 2.2. Osimertinib Suppressed Cell Cycle Progression Independent of EGFR Pathways

**Figure 2:**
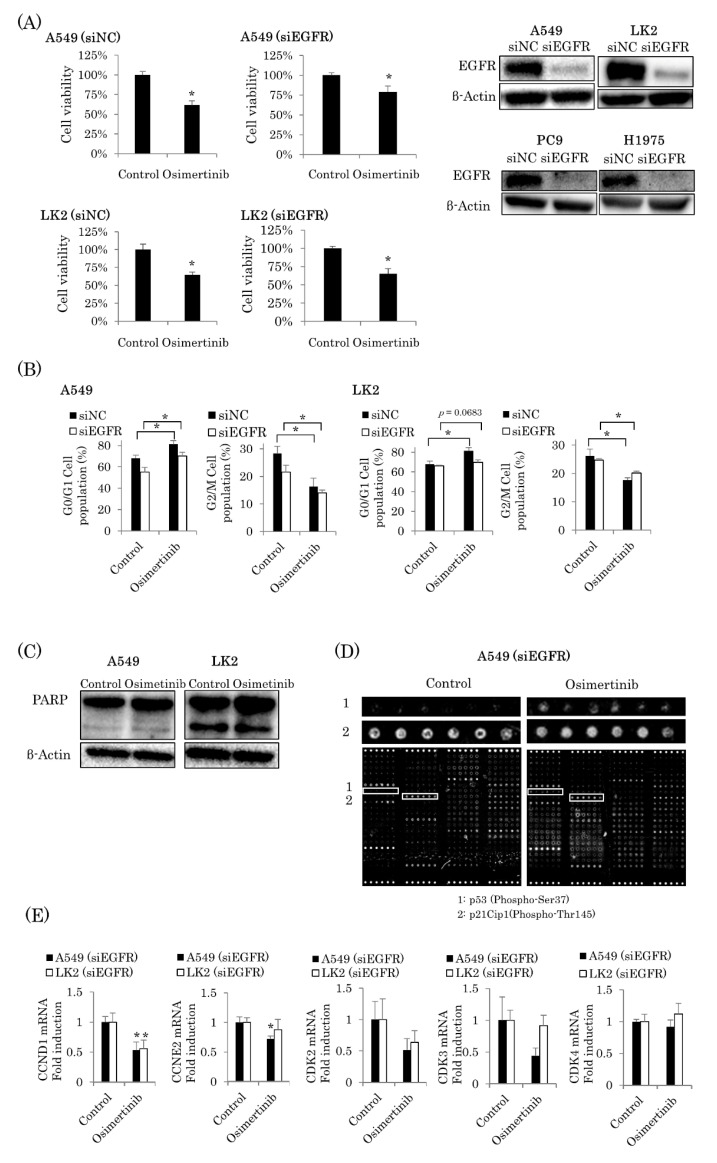
EGFR-independent inhibitory effects of osimertinib on cell proliferation. (**A**) Exposure to osimertinib for 72 h with siEGFR significantly decreased cell viability at IC50 in A549 and LK2 cells (*n* = 6). EGFR protein expression was decreased in all cell lines by siEGFR transfection for 48 h. (**B**) Osimertinib exposure at IC50 for 72 h with siNC or siEGFR increased G0/G1 phase, and decreased G2/M phase in both A549 and LK2 cells (*n* = 3). In EGFR knocked down LK2 cells, G0/G1 phase tended to increase (*n* = 3). (**C**) Exposure to osimertinib for 72 h at IC50 did not induce cleaved PARPin A549 or LK2 cells with siEGFR. (**D**) The results of the phosphorylation array regarding cell cycle regulators. Osimertinib exposure for 72 h at IC50 increased the expression of phosphorylated p21Cip1 and p53 in A549 cells with siEGFR (*n* = 6). (**E**) Osimertinib exposure for 72 h at IC50 with siEGFR significantly decreased the mRNA expression of CCND1 in A549 and LK2 cells (*n* = 3). The results were expressed as mean ± standard deviation. * *p* < 0.05.

To further explore the EGFR-independent growth inhibitory effects of osimertinib, we used EGFR knocked down EGFR wild-type NSCLC cell lines, A549 and LK2. Treatment with siEGFR and siRNA for negative control (siNC) at 1 nM (for A549 and LK2) or 5 nM (for PC9 and H1975) for 48 h had a knockdown effect on EGFR protein expression (Figure 2A). In both cell lines, osimertinib treatment at IC50 for 72 h significantly inhibited cell proliferation in EGFR knocked down cells (A549 and LK2 cells at 3 µM vs. control, all *p* < 0.0001) (Figure 2A). We subsequently performed cell cycle analyses using flow cytometry. Osimertinib exposure at IC50 for 72 h increased G0/G1 phase in A549 and LK2 cells (A549, siNC *p* = 0.0071, siEGFR *p* = 0.0096; LK2, siNC *p* < 0.0001, siEGFR *p* = 0.0683), although in EGFR knocked down LK2 cells the change did not reach a significant *p* value (*p* = 0.0683) (Figure 2B). The exposure decreased the G2/M phase in both A549 and LK2 cells (A549, siNC *p* = 0.0062, siEGFR *p* = 0.0100; LK2, siNC *p* = 0.0042, siEGFR *p* = 0.0011). In both the siNC and siEGFR transfected cell lines, osimertinib demonstrated the same effect on cell viability and cell cycle. Therefore, we only used siEGFR-transfected cell lines in following experiments.

Exposure to osimertinib for 72 h at IC50 did not induce the expression of cleaved poly(ADP-ribose) polymerase (PARP), a well-known marker of apoptosis, in EGFR knocked down A549 and LK2 (Figure 2C).

We then attempted to determine which cell cycle regulator could be involved in cell cycle suppression exerted by osimertinib. A phosphorylation array covering various cell cycle regulators was performed using A549 cells. Exposure to osimertinib for 72 h at IC50 increased the expression of phosphorylated p21Cip1 and p53 (Figure 2D). We studied the mRNA expression of factors well known to be related to the increase of G0/G1 phase: Cyclin (CCN) D1, CCNE2, Cyclin-dependent kinase (CDK) 2, CDK3, and CDK4. Consequently, the mRNA expression of CCND1 was significantly decreased in A549 and LK2 cells treated with osimertinib for 72 h at IC50 (CCND1, *p* = 0.0075 and *p* = 0.0214, respectively). CCNE2 was significantly reduced only in A549 cells (*p* = 0.0109). No significant changes in CDK2, CDK3, or CDK4 levels were detected in either cell line (Figure 2E).

### 2.3. EGFR-Independent Effect of Osimertinib on EphB4 in NSCLC

**Figure 3:**
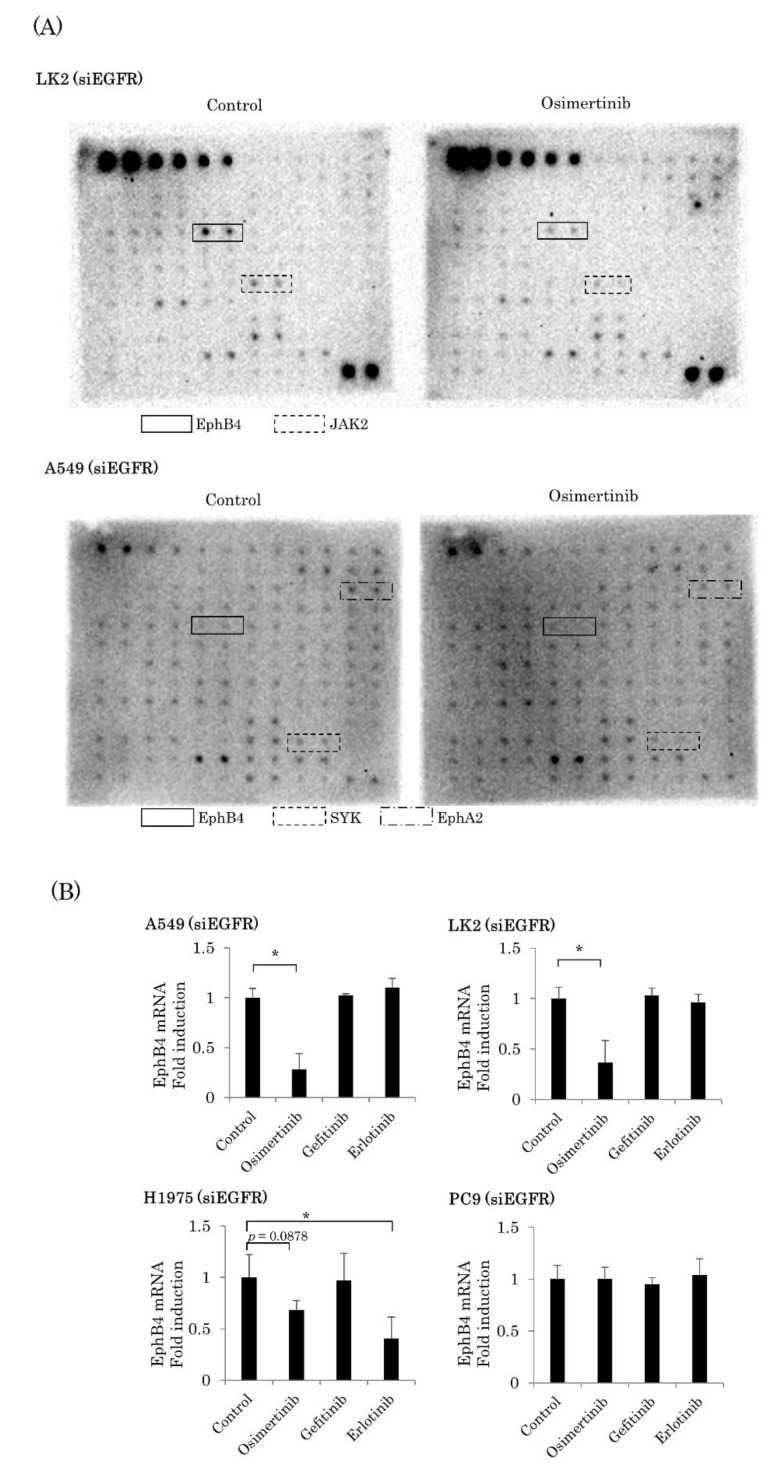
EGFR-independent suppression of EphB4 expression by osimertinib. (**A**) The results of the RTK phosphorylation antibody array. Osimertinib exposure for 72 h at IC50 with siEGFR suppressed the phosphorylation of EphB4 and JAK2 in LK2 cells and of EphB4, EphA2, and spleen tyrosine kinase (SYK) in A549 cells (*n* = 2). (**B**) Osimertinib exposure for 72 h at IC50 significantly decreased the mRNA expression of EphB4 in A549 and LK2 cells and tended to decrease that in H1975 cells (*n* = 3). Results were expressed as mean ± standard deviation. * *p* < 0.05.

To further explore which RTK of osimertinib acted on the suppression of cell cycle, a RTK phosphorylation array using EGFR knocked down A549 and LK2 cells was performed. After osimertinib exposure for 72 h, the suppression of the phosphorylation of EphB4, EphA2, and spleen tyrosine kinase was detected in A549, and that of EphB4 and Janus activating kinase 2 (JAK2) was detected in LK2 (Figure 3A). Therefore, we focused on EphB4 since it changed in multiple cell lines and had a higher phosphorylation level in the naive state than the others. We examined the association between osimertinib and total EphB4 in NSCLC using four EGFR knocked down cell lines: A549, LK2, PC9, and H1975. We exposed these cell lines to EGFR-TKIs for 72 h at IC50. We studied the mRNA expression of EphB4 in these four cell lines. Osimertinib exposure for 72 h at IC50 significantly decreased the mRNA expression of EphB4 in two cell lines (A549, *p* = 0.0007; LK2, *p* = 0.0013). In H1975 cells, osimertinib and erlotinib tended to decrease the mRNA expression of EphB4 (*p* = 0.0878 and *p* = 0.0280, respectively). In PC9, EphB4 expression was not altered by any EGFR-TKI. (Figure 3B).

### 2.4. Involvement of EphB4 in the EGFR-Independent Inhibitory Effects of Osimertinib

**Figure 4:**
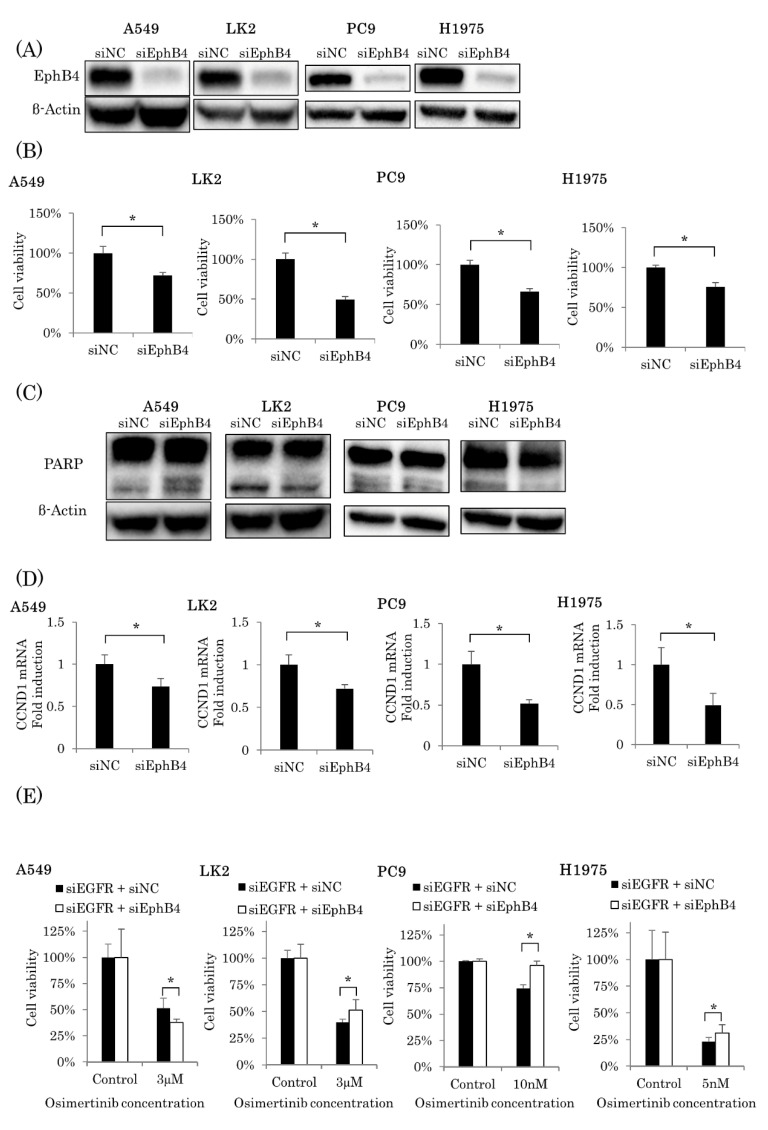
The association between EphB4 and the EGFR-independent growth inhibitory effects of osimertinib. (**A**) EphB4 protein expression in A549, LK2, PC9, and H1975 cells was knocked down by siEphB4 (5 nM) for 48 h. (**B**) In A549, LK2, PC9, and H1975 cells, cell proliferation was significantly inhibited by the knockdown of EphB4 (*n* = 6). (**C**) The induction of cleaved PARP was not induced by the knockdown of EphB4 in three of four cell lines. (**D**) The knockdown of EphB4 significantly decreased the mRNA expression of CCND1 in A549, LK2, PC9, and H1975 cells (*n* = 3). (**E**) The knockdown of EphB4 significantly reduced the EGFR-independent inhibitory effects of osimertinib on cell proliferation in LK2, PC9, and H1975 cells (*n* = 6). The results were expressed as mean ± standard deviation. * *p* < 0.05.

We studied the EGFR-independent effects of osimertinib and the correlation between EphB4 and cell proliferation. Figure 4A demonstrates the knockdown efficiency of siEphB4 using four cell lines: A549, LK2, PC9, and H1975. In all four cell lines, cell proliferation was significantly inhibited by the knockdown of EphB4 following the treatment with siEphB4 (5 nM) for 48 h (A549, *p* < 0.0001; LK2, *p* < 0.0001; PC9, *p* = 0.0001; H1975, *p* = 0.0001) (Figure 4B). In addition, apoptosis was not induced by the knockdown of EphB4 using siEphB4 (5 nM) for 48 h in three of four cell lines (Figure 4C). A significant decrease in CCND1 mRNA by the knockdown of EphB4 using siEphB4 (5 nM) for 48 h was also detected in all four cell lines (A549, *p* = 0.0348; LK2, *p* = 0.0181; PC9, *p* = 0.0072; H1975, *p* = 0.0276) (Figure 4D). However, CCND3 was significantly increased in three cell lines (A549, *p* = 0.0042; LK2, *p* < 0.0001; PC9, *p* < 0.0001).

We also studied the EGFR-independent growth inhibitory effects of osimertinib via EphB4 inhibition using EGFR and EphB4 double knocked down cell lines: A549, LK2, PC9, and H1975. The knockdown of EphB4 significantly reduced the EGFR-independent inhibitory effects on cell proliferation after osimertinib exposure at IC50 for 72 h in three of four cell lines (LK2, *p* = 0.0053; PC9, *p* < 0.0001; H1975, *p* = 0.0381) (Figure 4E).

### 2.5. EphB4 Was a Poor Prognostic Factor in EGFR Mutation-Positive Lung Adenocarcinoma

**Figure 5:**
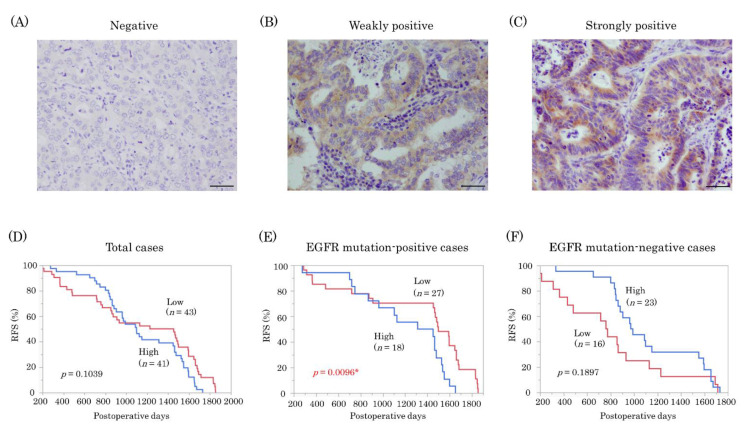
Immunohistochemistry of EphB4 in patients with lung adenocarcinoma. (**A**–**C**) Representative illustrations of immunohistochemistry of EphB4 in patients with lung adenocarcinoma (bar: 50 μm). EphB4 immunoreactivity was detected in both the cytoplasm and membrane of tumor cells: (**A**) negative, (**B**) weakly stained, and (**C**) markedly stained. (**D**–**F**) RFS in patients with high and low EphB4 expression. (**D**) RFS in all 84 cases. (**E**) RFS in 45 EGFR mutation-positive cases. (**F**) RFS in 39 EGFR mutation-negative cases. The high expression of EphB4 was a significant poor prognostic factor in EGFR mutation-positive cases. * *p* < 0.05.

The representative images of the results are illustrated in Figure 5A–C. EphB4 immunoreactivity was detected in the cytoplasm and membrane of the tumor cells. There were 43 cases tentatively defined as the low expression group and 41 cases as the high expression group. The correlation between EphB4 immunoreactivity and clinicopathological factors is summarized in Table 1. pT: pathological T factor; pN: pathological N factor; pStage: pathological Stage was evaluated based on the 7th edition UICC (Union for International Cancer Control) TNM system for lung tumors. EphB4 immunoreactivity was positively correlated with T factor (*p* = 0.0135), tumor size (*p* = 0.0027), and Ki-67 labeling index (LI) (*p* = 0.0004). The EGFR mutation-positive group was associated with a lower smoking index than the EGFR mutation-negative group (*p* = 0.0004).

Relapse-free survival (RFS) is presented in Figure 5D–F. No significant correlations were detected between EphB4 expression and prognosis (*p* = 0.1039) in all 84 cases (Figure 5D). In addition, the high expression of EphB4 was a significant adverse prognostic factor in EGFR mutation-positive cases (*n* = 45; *p* = 0.0096) (Figure 5E), whereas it was not a significant adverse prognostic factor in EGFR mutation-negative cases (*n* = 39; *p* = 0.1897) (Figure 5F).

## 3. Discussion

**Figure 6:**
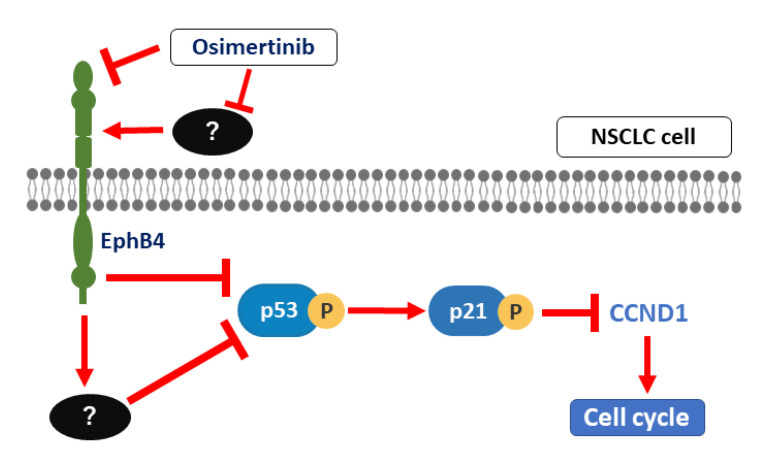
The outline of our hypothesis regarding the signal pathway of the anti-cancer effect of osimertinib via EphB4. Osimertinib may be involved in the suppression of cell proliferation via the EphB4 and p53-p21-Cyclin D1 pathway independent of the EGFR pathway in NSCLC cells.

In this study, we demonstrated for the first time that osimertinib may be involved in the suppression of cell proliferation via the EphB4 and p53-p21-Cyclin D1 pathway independent of the EGFR pathway in lung carcinoma (Figure 6). In addition, EphB4 had a significantly negative impact on the prognosis/clinical outcome in EGFR mutation-positive lung adenocarcinoma patients. These results indicate that EphB4 is a predictor of the therapeutic efficacy of osimertinib and of the subsequent clinical outcomes of patients with EGFR mutation-positive lung adenocarcinoma.

We explored pathways or molecules through which osimertinib could suppress cell proliferation independently of the EGFR pathway. Osimertinib exerted growth inhibitory effects on the EGFR mutation-positive lung carcinoma cell lines PC9 and H1975, even with the knockdown of EGFR, and on the EGFR mutation-negative lung carcinoma cell lines A549 and LK2 at high concentrations. In EGFR knocked down A549 and LK2 cell lines, osimertinib significantly increased G0/G1 phase and decreased G2/M phase. These results indicate that osimertinib could suppress the progression of cell cycle in lung cancer independent of the EGFR signaling pathway of carcinoma cells. In addition, the apoptosis-inducing effects of osimertinib were not necessarily apparent in the EGFR knocked down cell lines. Therefore, we postulated that the EGFR-independent cell growth inhibitory effects of osimertinib could be more markedly related to the suppression of cell cycle progression than to the induction of apoptosis. A subsequent comprehensive study of cell cycle progression using an antibody phosphorylation array revealed that osimertinib suppressed cell cycle progression via the inhibition of p53-p21-Cyclin D1 pathway independently of the EGFR pathway. The arrest of G1 phase and apoptosis induction are well known as important functions of p53, and it has been reported that these functions are exerted differently depending on the phosphorylation site of p53 [35]. In general, marked DNA damage is known to induce the phosphorylation of the 46th serine residue of p53, resulting in the increased expression of the p53-regulated apoptosis-inducing protein 1 followed by apoptosis, and vice versa [36]. In the present study, osimertinib increased the phosphorylation level of p53 only at the Ser37 site. This was considered one of the reasons why osimertinib did not significantly induce apoptosis in our present study.

We then focused on EphB4 as a factor involved in the EGFR-independent p53-p21-Cyclin D1 pathway suppression of osimertinib using an RTK phosphorylation antibody array. EphB4 has been reported to be a poor prognostic factor in NSCLC and lung adenocarcinoma patients [37,38]. Furthermore, knockdown of EphB4 has been reported to suppress the cell proliferation of lung adenocarcinoma cell lines [39], and knockdown of EphA2 has been reported to improve gefitinib sensitivity in gefitinib-resistant lung adenocarcinoma cell lines [40]. Therefore, EphB4 could play an important role in tumor growth in lung cancer, but the association between EphB4 and EGFR mutations or EGFR-TKIs in lung cancer has not been reported thus far, to the best of our knowledge.

Therefore, in this study, we examined the association between EphB4 and cell proliferation both in vitro and in immunohistochemical studies using human lung adenocarcinoma tissues. Knockdown of EphB4 inhibited cell proliferation in both EGFR mutation-positive and -negative lung carcinoma cell lines, consistent with previous studies [39]. In addition, knockdown of EphB4 decreased the expression of CCND1 but did not induce apoptosis. The overexpression of CCND1 in NSCLC has been reported to be associated with cell proliferation and tumorigenesis [41,42]. The results of these studies indicated that CCND1 expression levels could influence the growth of tumor cells in NSCLC. Therefore, EphB4 was reasonably postulated to promote cell proliferation via increased CCND1. Immunohistochemical studies using human lung adenocarcinoma demonstrated that the EphB4 status in carcinoma cells was positively correlated with tumor size, T factors, and Ki-67 LI, all of which represented increased cell growth ability. These results were consistent with the results of our present in vitro investigations. Furthermore, EphB4 turned out to be a significant poor prognostic factor in EGFR mutation-positive lung adenocarcinoma patients but not in EGFR mutation-negative patients. EGFR mutation-positive lung adenocarcinoma is well-known as a non-smoker type, whereas EGFR mutation-negative lung adenocarcinoma is a smoker type, and smoking has been reported to induce a high tumor mutation burden [43]. Therefore, we hypothesized that the lower impact of EphB4 on cell proliferation in EGFR mutation-negative lung adenocarcinoma could be due to the potential influence of various other factors related to smoking. In addition, Eph has been reported to suppress the wild type of EGFR pathway [44], whereas in patients with EGFR-mutation, the EGFR-independent tumor growth-promoting effects may outweigh the inhibitory effects of EphB4. Therefore, EphB4 may be a significant prognostic factor in patients with EGFR mutations. However, further investigations are required to clarify these points.

The association between osimertinib therapy and EphB4 also demonstrates that osimertinib decreased the expression of EphB4 and that the knockdown of EphB4 reduced the sensitivity of osimertinib in lung carcinoma cell lines regardless of the EGFR mutation status. These results also suggest that osimertinib can suppress cell cycle in an EGFR-independent manner via the downregulated EphB4 and p53-p21-Cyclin D1 pathways. p53 protein suppresses the expression of EphB4 [45,46]. Therefore, EphB4 and the p53-p21-Cyclin D1 pathway may interact with each other. However, further experiments are needed to demonstrate that osimertinib has EGFR-independent inhibitory effects on cell cycle progression mediated by the interaction between EphB4 and the p53-p21-Cyclin D1 pathways using multiple cell lines, although only A549 was used in the antibody microarray analysis of the phosphorylation of cell cycle factors.

In summary, this is the first report to reveal that osimertinib has EGFR-independent inhibitory effects on cell cycle progression mediated by the interaction between EphB4 and the p53-p21-Cyclin D1 pathways and that EphB4 is a significantly poor prognostic factor, especially in EGFR mutation-positive lung adenocarcinoma. The results of our present study can contribute to the development of effective therapeutic strategies for osimertinib in EGFR mutation-positive lung adenocarcinoma cases by focusing on the characteristics of EphB4 as a predictor of the therapeutic effects of osimertinib and as a prognostic factor. Osimertinib can exert stronger growth inhibitory effects in NSCLC patients with a high expression of EphB4 than in those with a low expression of EphB4, and it may improve the prognosis in EGFR mutation-positive lung adenocarcinoma patients.

## 4. Material and Methods

### 4.1. Reagents and Antibodies

The following materials were commercially obtained: osimertinib (Chemscene, Monmouth Junction, NJ, USA), gefitinib (Wako Pure Chemical Industries, Osaka, Japan), erlotinib (Wako Pure Chemical Industries), and DMSO (Wako Pure Chemical Industries). Antibodies were obtained from the following sources: EGFR [EP38Y] (Abcam, Cambridge, UK), PARP [46D11] (Cell Signaling Technology, Beverly, MA, USA), EphB4 [D1C7N] (Cell Signaling Technology), β-actin [AC-15] (Sigma-Aldrich, Burlington, MO, USA), and Ki-67 [MIB1] (DAKO, Santa Clara, CA, USA).

### 4.2. Cell Culture

The following human NSCLC cell lines were used in this study: A549 (Health Science Research Resources Bank, Osaka, Japan), LK2 (Cell Resource Centre for Biomedical Research, Miyagi, Japan), PC9 (Riken Cell Bank, Tsukuba, Japan), and NCI-H1975 (H1975) (American Type Culture Correction, Manassas, VA, USA). A549, PC9, and H1975 are lung adenocarcinoma cell lines, and LK2 is a lung squamous cell carcinoma cell line. The EGFR mutations in these cell lines are as follows: A549 and LK2; EGFR wild-type, PC9; exon 19 deletion, H1975; and T790M and L858R mutation. The cells were maintained in Roswell Park Memorial Institute media (RPMI) 1640 (Sigma-Aldrich) supplemented with 10% fetal bovine serum (FBS) (Nichirei, Tokyo, Japan) incubated at 37 °C with 5% CO_2_.

### 4.3. Cell Viability Assays

Each cell line (5000 cells/well) was seeded in 96-well plates and incubated in an RPMI-1640 medium containing 10% FBS. After preincubation and/or siRNA transfection for 48 h, the cells were treated with each reagent for 72 h at concentrations ranging from 3 pM to 10 µM, resulting in a total of 120 h of transfection time. DMSO was used as a control. Cell viability was measured using the WST-8 Cell Counting Kit-8 (Dojindo Laboratories, Kumamoto, Japan).

### 4.4. Western Blotting

Total protein was extracted from cultured cells using an M-PER Mammalian Protein Extraction Reagent (Thermo Fisher Scientific, Waltham, MA, USA). The protein concentration was measured using the Protein Assay Rapid Kit Wako (Wako Pure Chemical Industries), and the total protein was individually subjected to sodium dodecyl sulfate polyacrylamide gel electrophoresis (SuperSep Ace, Wako Pure Chemical Industries). These proteins were subsequently transferred onto a Trans-Blot Turbo Transfer Pack Mini format 0.2 µm polyvinylidene fluoride membrane (Bio-Rad, Hercules, CA, USA), and blotting was performed using the Trans-Blot Turbo transfer system (Bio-Rad). The proteins on the membrane were blocked in 5% nonfat dry skim milk powder (Wako Pure Chemical Industries) in Tris buffered saline with Tween 20 for 1 h at room temperature and were incubated with primary antibodies overnight at 4 °C. The primary antibodies were diluted using an Immuno Shot (Cosmo Bio, Tokyo, Japan) as follows: EGFR, 1:1000; EphB4, 1:1000; cleaved PARP, 1:1000; and β-actin, 1:5000. These antibody-protein complexes were detected using ECL-plus western blotting detection reagents (GE Healthcare, Chicago, IL, USA) after incubation with anti-rabbit or anti-mouse IgG secondary antibodies at room temperature (15–25 °C) for 1 h. The protein bands were visualized using the ChemiDoc XRS+ System (Bio-Rad) and Image Lab version 5.0 software (Bio-Rad).

### 4.5. RNA Interference

The siRNAs targeting EGFR and EphB4, which were used for the gene knockdown, were purchased from GeneDesign, Inc. (Osaka, Japan) and were as follows: siEGFR sense, 5′-CAAAGUGUGUAACGGAAUATT-3′; siEGFR antisense, 5′-UAUUCCDUUACACACUUUGTT-3′; siEphB4 sense, 5′-GGUGAAUGUCAAGACGCUGTT-3′; and siEphB4 antisense, 5′-CAGCGUCUUGACAUUCACCTT-3′. Silencer Select Negative Control 1 siRNA (Ambion, Austin, TX, USA) was used as a negative control (siNC). The siRNA was transfected into cells (1 × 10^5^ cells/mL) using an Opti-MEM^™^ I Reduced Serum Medium (Thermo Fisher Scientific) and a Lipofectamine RNAi MAX reagent (Invitrogen, Carlsbad, CA, USA). The concentrations were as follows: siEGFR and siNC, 1 nM for A549 and LK2 and 5 nM for PC9 and H1975; and siEphB4 and siNC, 5 nM for all four cell lines. The transfection time was 72 h, but when EGFR-TKI treatment was performed under siRNA transfection, we transfected siRNA at 48 h before EGFR-TKI treatment for 72 h, resulting in a total of 120 h of transfection time.

### 4.6. Cell Cycle Analysis

Cell cycle was analyzed according to the manufacturer’s protocols (Becton Dickinson, Franklin Lakes, NJ, USA). After treating siEGFR-transfected A549 and LK2 cells with osimertinib at IC50 (3 µM each) or DMSO for 72 h, the cells were washed twice with phosphate-buffered saline (PBS), detached with Accutase (Stem cell, Vancouver, Canada), and centrifuged. We added 70 % ethanol (Wako Pure Chemical Industries) stored at −20 °C to the cells, and the mixture was incubated at −20 °C for 2 h. After washing twice with PBS and centrifugation, the cells were incubated in 0.5 mL of PI/RNase staining buffer (Becton Dickinson) at room temperature for 15 min. Cell cycle was analyzed by FACS Aria III (Becton Dickinson). The data were analyzed to determine the percentage of cells in the G0/G1 and G2/M phases.

### 4.7. Antibody Microarray Analysis of Phosphorylation of Cell Cycle Factors

This array was performed according to the manufacturer’s protocol (Full Moon Biosystems, Sunnyvale, CA, USA). Briefly, after the treatment of siEGFR-transfected A549 cells with osimertinib at IC50 (3 µM) or DMSO for 72 h, the total protein was extracted from the cells using a Lysis Buffer (Full Moon Biosystems). The protein was subsequently biotinylated and placed on pre-blocked microarray slides of a Cell Cycle Phospho Antibody Array [Cat. No. PCC076] (Full Moon Biosystems). We then detected phosphorylated proteins using Cy3-conjugated streptavidin (BioLegend, San Diego, CA, USA). The slides were scanned using a Microarray Scanner (Agilent Technologies, Santa Clara, CA, USA).

### 4.8. Real Time RT-PCR

The total RNA was extracted from cultured cells using a TRI reagent (Molecular Research Center, Cincinnati, OH, USA) and was reverse transcribed to cDNA using a QuantiTect Reverse Transcription Kit (Qiagen, Hilden, Germany). The mRNA expression was semiquantified using real-time RT-PCR in a LightCycler System (Roche Diagnostics, Mannheim, Germany).

The PCR mixture (20 μL) included 1 μM primer and FastStart Essential DNA Green Master (Roche Diagnostics). The PCR protocol was as follows: initial denaturation at 95 °C for 5 min, 40 amplification cycles at 95 °C for 10 s, and annealing at 60 °C for 30 s. The following primer sequences were used in this study: CCND1 forward, 5′-ATGTTCGTGGCCTCTAAGATGA-3′; CCND1 reverse, 5′-CAGGTTCCACTTGAGCTTGTTC-3′; CCNE2 forward, 5′-CTATTTGGCTATGCTGGAGG-3′; CCNE2 reverse, 5′-TCTTCGGTGGTGTCATAATG-3′; CDK2 forward, 5′-AGCCAGAAACAAGTTGACGG-3′; CDK2 reverse, 5′-TGATGAGGGGAAGAGGAATG-3′; CDK3 forward, 5′-GTTTCTGCCACTCACATCGG-3′; CDK3 reverse, 5′-ACCACAGTGTCACCACCTCAT-3′; CDK4 forward, 5′-TTGCATCGTTCACCGAGATC-3′; CDK4 reverse, 5′-CTGGTAGCTGTAGATTCTGGCCA-3′; EGFR forward, 5′-TGGTAGCACTTGCTACCCTGAGTTCAT-3′; EGFR reverse, 5′-TGGGCTGGAATCCGAGTTATTATTTGATGT-3′; EphB4 forward, 5′-GTCTGACTTTGGCCTTTC-3′; EphB4 reverse, 5′-TGACATCACCTCCCACATCA-3′; RPL13A forward, 5′-CCTGGAGGAGAAGAGGAAAG-3′; and RPL13A reverse, 5′-TTGAGGACCTCTGTGTATTT-3′. The mRNA levels of each gene were expressed as the ratio between the housekeeping gene and the RPL13A mRNA levels.

### 4.9. Human Receptor Tyrosine Kinase Phosphorylation Antibody Array

The total protein was extracted using an M-PER Mammalian Protein Extraction Reagent (Thermo Fisher Scientific) from siEGFR-transfected LK2 cells exposed to osimertinib at IC50 (3 µM) for 72 h. The array was conducted using Human RTK Phosphorylation Antibody Arrays (Ray Biotech, Peachtree Corners, GA, USA) according to the manufacturer’s protocol. For detection, we used Image Lab version 5.0 software (Bio-Rad).

### 4.10. Patients and Tissue Preparation

A total of 84 lung adenocarcinoma cases were examined in this study. Specimens were retrieved from patients who underwent surgical resection from 2014 to 2015 at the Department of Thoracic Surgery, Miyagi Cancer Center, Miyagi, Japan. None of the patients received chemotherapy or irradiation prior to surgery. The study was conducted in accordance with the Declaration of Helsinki. The protocol was approved by the ethics committee at the Tohoku University School of Medicine and the Miyagi Cancer Center, and all patients provided informed consent.

### 4.11. Immunohistochemistry

We used rabbit monoclonal antibodies against EphB4 and Ki-67, and the Histofine Kit (Nichirei) was used based on the streptavidin–biotin amplification method. The dilutions of the primary antibodies were 1:100 for both EphB4 and Ki-67. Human colon carcinoma and tonsils were used as positive controls for EphB4 and Ki-67, respectively. The antigen retrieval of EphB4 and Ki-67 was performed using an autoclave treatment at 121 °C for 5 min in pH 9 buffer for EphB4 and citric buffer for Ki-67. Antigen–antibody complexes were visualized using 3,3′-diaminobenzidine (DAB) solution (1 mM DAB, 50 mM Tris–HCl buffer pH 7.6, and 0.006% H_2_O_2_) and counterstained with hematoxylin.

We evaluated the immunoreactivity of the whole tissue slide of all the cases examined in this study. Cells demonstrating higher immunointensity than the background were tentatively defined as positive in this study. A modified histological score (H-score) was used to evaluate EphB4. The H-score was obtained by adding the percentage of strongly stained tumor cells (2×) with that of weakly stained tumor cells (1×), providing a possible range from 0 to 200. The median modified H-score was 110; therefore, we defined ≥110 as the high expression group and <110 as the low expression group. The proliferative activity of the tumor cells was assessed using the Ki-67 LI, which was calculated for the area with the highest percentage of positive nuclei (hot spot) based on the counting at least 500 tumor cells. All slides were independently examined by RN and RS.

### 4.12. Statistical Analysis

Statistical analyses were performed using JMP Pro 15 (SAS Institute, Cary, NC, USA). The statistical differences between the two groups were evaluated using Student’s *t*-test or the chi-square test with a generalized Fisher’s test, as appropriate. RFS curves were generated according to the Kaplan–Meier method. Statistical significance was set at *p* < 0.05.

## Figures and Tables

**Table 1:** The association between EphB4 immunoreactivity and clinicopathological factors in patients with lung adenocarcinoma (*n* = 84).

	EphB4 Immunoreactivity		
	Low (*n* = 43)	High (*n* = 41)	*p* Value
Age	67.5 ± 9.55	65.3 ± 9.41	0.2920
Sex			0.2639
Male	21	25	
Female	22	16	
Smoking Index	371.8 ± 521.7	482.8 ± 597.2	0.3665
pStage			0.6505
I	30	25	
II	4	6	
III	9	10	
pT			0.0135 *
1	28	14	
2	14	23	
3	1	4	
pN			0.7048
0	34	31	
1–3	9	10	
Tumor size	23.14± 11.93	31.27± 12.13	0.0027 *
Ki-67 Labeling index (%)	4.58± 5.61	13.14± 13.27	0.0004 *
EGFR mutation			0.0827
Positive	27 (Ex19Del: 14, L858R: 12, Ex19Del & L858R: 1)	18 (Ex19Del: 8, L858R: 7, Ex19Del & L858R: 2, G719X: 1)	
Negative	16	23	

Data are presented as average ± standard deviation, * *p* value < 0.05 significant. pT: pathological T factor; pN: pathological N factor; pStage: pathological Stage, based on the 7th edition UICC (Union for International Cancer Control) TNM system for lung tumors; EGFR: epidermal growth factor receptor.

## Data Availability

All data generated or analyzed during this study are included in this published article.

## Abbreviations

ALK	anaplastic lymphoma kinase
CCN	Cyclin
CDK	Cyclin-dependent kinase
DMSO	Dimethyl sulfoxide
EGFR	epidermal growth factor receptor
EGFR-TKI	EGFR-tyrosine kinase inhibitor
Eph	ephrin receptor
FBS	fetal bovine serum
HER2	Human EGFR-related 2
JAK2	Janus activating kinase 2
LI	labelling index
NSCLC	non-small cell lung cancer
PARP	poly(ADP-ribose) polymerase
PBS	phosphate-buffered saline
RFS	Relapse-free survival
RPMI	Roswell Park Memorial Institute media
TKI	tyrosine kinase inhibitor
